# LRP1 in atherosclerosis: a hierarchical view of regulatory mechanisms and epigenetic knowledge gaps

**DOI:** 10.3389/fcell.2026.1829891

**Published:** 2026-05-08

**Authors:** Rocio Casale, Gustavo A. Chiabrando, Danilo G. Ceschin

**Affiliations:** 1 Hospital Privado Universitario de Córdoba, Córdoba, Argentina; 2 Instituto Universitario de Ciencias Biomédicas de Córdoba (IUCBC), Córdoba, Argentina; 3 Centro de Investigación en Medicina Traslacional “Severo R. Amuchástegui” (CIMETSA), U.A. Consejo Nacional de Investigaciones Científicas y Técnicas (CONICET), Córdoba, Argentina

**Keywords:** atherosclerosis, epigenetic regulation, LRP1, macrophage plasticity, VSMC

## Abstract

Inflammation is a central driver of vascular dysfunction and plays a pivotal role in the initiation and progression of atherosclerosis. This chronic inflammatory condition arises from complex molecular, cellular, and epigenetic interactions within vascular and immune networks. Monocytes and macrophages act as key mediators of plaque development, integrating lipid handling and inflammatory signaling. Low-density lipoprotein receptor-related protein 1 (LRP1) has emerged as a multifunctional receptor at the interface between lipid metabolism and immune regulation, modulating Toll-like receptor pathways and inflammatory responses in a context-dependent manner. Here, we review current evidence on the regulatory mechanisms controlling LRP1 expression and propose a hierarchical framework in which transcription factor-mediated and non-coding RNA-mediated mechanisms represent the most strongly supported layers of regulation. In contrast, although epigenetic processes broadly influence vascular inflammation, direct chromatin-level interrogation of the LRP1 locus remains limited. Together, this framework highlights key knowledge gaps and provides a conceptual basis for understanding how multi-layered regulation of LRP1 contributes to vascular inflammation and plaque progression.

## 1 Introduction

Atherosclerosis (AS) is now recognized as a chronic inflammatory disease driven by lipid accumulation, metabolic stress, and innate immune activation within the vascular wall (Chen et al., 2021; Sizova et al., 2023). During lesion development, circulating monocytes infiltrate the intima and differentiate into macrophages that regulate inflammatory amplification or resolution depending on local environmental signals (Libby et al., 2002; Ijaz et al., 2025). In parallel, vascular smooth muscle cells (VSMC) undergo phenotypic modulation, contributing to extracellular matrix remodeling, lipid accumulation, and plaque stability (Bennett et al., 2016). The dynamic interplay between macrophages and VSMC critically shapes lesion progression and structural integrity. Macrophage plasticity, traditionally described along a pro-inflammatory (M1-like) to reparative (M2-like) spectrum, critically influences plaque progression, and epigenetic mechanisms contribute to stabilizing or reprogramming these activation states within lesions (Yang et al., 2022).

Low-density lipoprotein receptor-related protein 1 (LRP1) has emerged as a multifunctional molecule at the interface between lipid metabolism and immune regulation. Structurally, LRP1 is a large endocytic and signaling receptor composed of extracellular ligand-binding domains and an intracellular tail containing motifs that recruit adaptor proteins involved in endocytosis, vesicular trafficking, and signal transduction (Barcelona et al., 2013; Gonias and Campana, 2014; Jaldín-Fincati et al., 2019; Yamamoto et al., 2024). LRP1 is widely expressed in VSMC, monocytes, and macrophages, where it contributes to lipid uptake, clearance of protease-inhibitor complexes, and extracellular matrix homeostasis (Actis Dato and Chiabrando, 2018; Chen et al., 2021). Beyond these canonical roles, LRP1 modulates innate immune signaling by influencing Toll-like receptor (TLR)-dependent pathways, particularly TLR2 and TLR4, thereby affecting NF-κB activation and cytokine production (Mantuano et al., 2016; Luo et al., 2018). These effects are context-dependent and shaped by ligand availability, receptor abundance, and cellular state rather than representing uniform suppression of inflammatory signaling.

Recent data indicate that LRP1 expression and activity are dynamically regulated under inflammatory and lipid-rich conditions. Reduced LRP1 transcription and protein levels in peripheral blood monocytes (PBM) from individuals with subclinical AS have been associated with a pro-inflammatory phenotype, highlighting potential translational relevance (Albertini et al., 2022). The mechanisms controlling LRP1 expression across different cellular and pathological contexts remain incompletely understood. In this review, we integrate current evidence on the regulation of LRP1 expression at different levels, with a focus on transcriptional control, non-coding RNA-mediated mechanisms, and emerging chromatin-level regulatory processes. We further highlight key knowledge gaps and propose a framework to understand how multi-layered regulation of LRP1 contributes to vascular inflammation and plaque progression.

## 2 Structural and functional features of LRP1

LRP1 is a type I transmembrane receptor of the LDL receptor family composed of a large extracellular α-subunit (∼515 kDa) and a transmembrane β-subunit (∼80–85 kDa). This molecular structure enables LRP1 to function as both a high-capacity endocytic receptor and a signaling platform, and it has been broadly reviewed (Lillis et al., 2008; Strickland et al., 2014; Yamamoto et al., 2024). LRP1 contributes to lipid handling, extracellular matrix turnover, and cellular homeostasis through the internalization of diverse ligands, including protease complexes, extracellular matrix components and modified lipoproteins (Mao et al., 2017; Actis Dato et al., 2018; Jaldín-Fincati et al., 2019).

In VSMC, LRP1 maintains vascular integrity by modulating extracellular matrix turnover and phenotypic stability (Lillis et al., 2008; Strickland et al., 2014; Chen et al., 2021). In monocytes and macrophages, it regulates lipid uptake, efferocytosis, and inflammatory signaling (Yancey et al., 2010; Mantuano et al., 2016). Myeloid-specific deletion of LRP1 accelerates AS in experimental models, supporting a protective role in macrophage-driven inflammation (Yancey et al., 2011). Conversely, its ability to mediate uptake of modified lipoproteins links LRP1 to foam cell formation under lipid-rich conditions, underscoring its context-dependent function. In macrophages, LRP1 regulates intracellular signaling pathways, including PI3K/Akt and MAPK/ERK, and regulates innate immune responses through interactions with pattern-recognition receptors such as TLRs, influencing NF-κB-dependent cytokine production (Gonias and Campana, 2014; Strickland et al., 2014; Mantuano et al., 2016). Simultaneously, TLR activation can induce tyrosine phosphorylation within the LRP1 intracellular domain, promoting recruitment of the Rab8a-PI3Kγ complex and subsequent Akt/mTOR activation. These signaling pathways may contribute to macrophage reprogramming and biased cytokine output that limits excessive inflammatory responses (Luo et al., 2018).

## 3 Regulation of LRP1 expression and function

**FIGURE 1:**
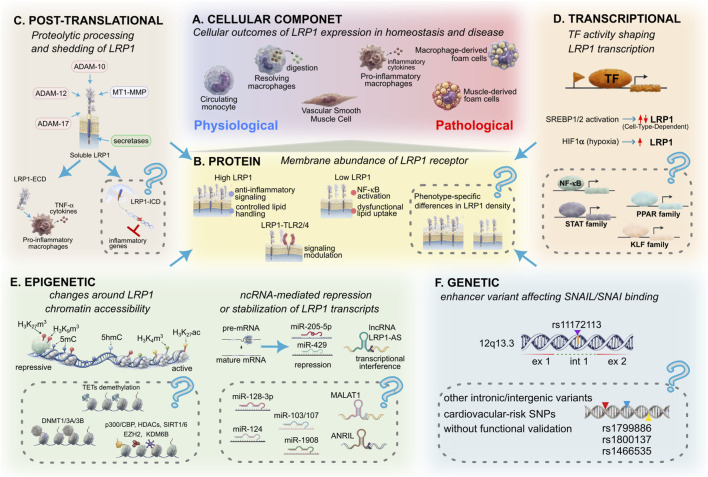
Multilevel regulation of LRP1 expression and function. Schematic representation of the multilevel regulatory mechanisms controlling LRP1 expression and their impact on cellular phenotypes within the vascular microenvironment. Cellular outcomes associated with differential LRP1 expression are represented in panel **(A)**, ranging from homeostatic states to pro-inflammatory and foam-cell-like phenotypes during AS progression. Regulation at the protein level **(B)** reflects how differences in membrane LRP1 abundance influence signaling and lipid handling. Post-translational proteolytic processing and receptor shedding mediated by intra- and extracellular proteases regulate cell surface abundance and signaling properties of LRP1 **(C)**. Transcriptional control is mediated by metabolic and inflammatory transcription factors **(D)**, while epigenetic mechanisms involving chromatin organization, DNA/histone modifications, and post-transcriptional regulation by non-coding RNAs contribute to LRP1 regulation **(E)**. Genetic variants and enhancer-associated elements further influence transcriptional regulation of LRP1 **(F)**. Validated pathways are shown in color; speculative mechanisms are indicated by dotted grey boxes with a “(?)”.

LRP1 expression is regulated through interconnected mechanisms that enable dynamic adaptation to physiological and pathological conditions (Yamamoto et al., 2024). Despite sharing the same genome, vascular and immune cells deploy distinct epigenetic programs that shape cell-type-specific transcriptional responses to lipid exposure, inflammatory cues, and environmental stressors. Metabolic signaling, transcription factor activity, post-transcriptional regulation, and chromatin-associated mechanisms collectively modulate LRP1 expression (Actis Dato and Chiabrando, 2018; Soehnlein and Libby, 2021; Sizova et al., 2023). However, how these regulatory layers converge to shape LRP1 expression during AS progression remains incompletely defined. Figure 1 summarizes this multilevel regulatory framework, illustrating how post-translational processing, transcriptional regulation, epigenetic mechanisms, and genetic variation collectively influence LRP1 expression and function. Together, these layers indicate that LRP1 expression emerges from coordinated interactions across multiple regulatory layers rather than isolated pathways.

### 3.1 Abundance and post-translational processing

LRP1 levels and function are regulated through multiple mechanisms controlling cell surface receptor abundance, subcellular localization, and post-translational processing, which is dynamically adapted to local metabolic and inflammatory conditions. In macrophages, exposure to modified LDL, including aggregated and oxidized LDL, has been associated with increased LRP1 expression, NF-κB activation, and foam cell formation (Llorente-Cortés et al., 2002; 2007; Costales et al., 2013; Vazquez et al., 2020). Membrane LRP1 cell surface abundance may also influence TLR2 and TLR4 signaling thresholds, potentially modulating macrophage polarization along the M1-M2 spectrum. In VSMC, aggregated LDL promotes LRP1 upregulation while repressing LDL receptor expression, favoring cholesteryl ester accumulation, enhanced proliferative and migratory capacity, and acquisition of muscle-derived foam cell phenotypes (Figures 1A,B) (Boucher and Herz, 2011; Solanelles Curco et al., 2025).

The cell surface LRP1 levels are regulated by intra- and extracellular proteolytic shedding mediated by ADAM family members, membrane-type matrix metalloproteinases (MT-MMPs), and secretases (Figure 1C) (Von Arnim et al., 2005; Liu et al., 2009; Gorovoy et al., 2010). These proteases are increased in the vascular intima during plaque development (Hua and Nair, 2015) and may influence receptor turnover, signaling capacity, and promotion of M1-like phenotypes. Shedding of the LRP1 α-Subunit generates soluble LRP1 (sLRP1-ECD), which modulates inflammatory responses in macrophages (Gorovoy et al., 2010). Elevated circulating sLRP1 levels correlate with atherosclerotic lesions, suggesting potential biomarker relevance (Garcia et al., 2023). However, whether sLRP1 exerts pro-inflammatory, buffering, or context-dependent effects *in vivo* remains unresolved. Regulated intramembrane proteolysis of LRP1 generates an intracellular domain (LRP1-ICD), which has been proposed to participate in intracellular signaling and potentially in transcriptional regulation (May et al., 2002). Although no direct evidence supports a similar role in AS, these findings raise the possibility that regulated intramembrane proteolysis influences transcriptional programs in vascular and immune cells.

In addition to proteolytic shedding, intracellular proteostasis mechanisms represent an additional layer of post-translational regulation. LRP1 is subject to proteasomal degradation, supporting a role for ubiquitin-dependent turnover in the regulation of receptor stability (Ceschin et al., 2009; Cáceres et al., 2010). Beyond LRP1, ubiquitin-mediated regulation has emerged as an important mechanism controlling protein stability and inflammatory signaling in vascular disease, with ubiquitin-specific proteases (USPs) acting as key modulators of macrophage activation, lipid metabolism, and vascular remodeling (Khan et al., 2026). At the level of AS, ubiquitin-dependent mechanisms directly influence disease progression, as illustrated by SCF E3 ligase-mediated ubiquitination of NLRP3, which regulates inflammasome activation and macrophage pyroptosis (Wang et al., 2025). However, whether similar ubiquitin-dependent mechanisms directly control LRP1 turnover and function in AS remains to be determined.

### 3.2 Transcriptional regulation

Sterol regulatory element-binding proteins (SREBPs) represent a key axis linking lipid metabolism to LRP1 expression (Figure 1D). SREBP-1 and SREBP-2 regulate cholesterol homeostasis through sterol-responsive elements within the LRP1 promoter. In VSMC, SREBP-2 negatively modulates LRP1 transcription, whereas aggregated LDL reduces SREBP-2 promoter binding, increasing receptor expression (Costales et al., 2010). In macrophages, inflammatory stimuli may alter SREBP activity, and reduced LRP1 transcription has been observed under pro-inflammatory conditions (Llorente-Cortés et al., 2006), although the precise mechanisms remain to be clarified.

Hypoxia, a feature of advanced atherosclerotic plaques, provides an additional regulatory input. Stabilization of HIF-1α under hypoxic conditions increases LRP1 transcription in VSMC by binding hypoxia response elements within the promoter, promoting aggregated LDL uptake and lipid accumulation that contribute to foam cell-like phenotypes and vascular remodeling. Hypoxia may also modulate SREBP activity, suggesting integration of metabolic stress pathways in LRP1 regulation (Dickson et al., 2023; Nakahara et al., 2023). Whether the HIF-1α-LRP1 axis operates similarly in macrophages remains unclear.

Regarding other transcription factors central to inflammatory signaling, such as NF-κB, PPARs, STATs, and KLFs, coordinate macrophage activation, lipid metabolism, and vascular remodeling, suggesting potential direct or indirect effects on LRP1 transcription (Figure 1D) (Zheng et al., 2024). However, direct promoter-level regulation of LRP1 by these factors has not been conclusively demonstrated. In addition, CREB-associated signaling emerged as a key regulator of gene expression in AS, integrating extracellular cues with transcriptional responses (Chen et al., 2023). Although no direct regulatory mechanism specific to LRP1 has been demonstrated, CREB-related mechanisms provide relevant upstream context for understanding how transcription factor networks may influence LRP1 expression under inflammatory conditions. To provide a structured overview of the current evidence, transcriptional regulators of LRP1 are summarized in Supplementary Table S1, highlighting differences across cell types and distinguishing between direct and indirect regulatory mechanisms.

Together, these observations indicate that transcription factor-mediated regulation of LRP1 is supported by varying levels of evidence, with direct promoter-level mechanisms established for selected factors, while others remain inferred from broader inflammatory and metabolic regulatory networks.

### 3.3 Non-coding RNAs

Several ncRNAs have been shown to directly target LRP1 mRNA, providing mechanistic evidence for its regulatory role in vascular and immune contexts. Non-coding RNA-mediated mechanisms currently provide some of the most direct evidence linking molecular regulation to changes in LRP1 expression (Figure 1E).

#### 3.3.1 Direct regulators of LRP1

MicroRNAs regulate gene expression through RNA-induced silencing complex (RISC)-mediated binding to complementary sequences within the 3′untranslated region (3′UTR) of target transcripts, resulting in mRNA destabilization or translational repression. MicroRNAs (Supplementary Table S2), such as miR-205-5p, directly bind the LRP1 3′UTR reducing receptor expression and impairing cholesterol efflux, particularly under oxidative or lipid-rich conditions. This repression disrupts the LRP1/ABCA1 axis, contributing to lipid accumulation and inflammatory activation in macrophages (Song and Bu, 2009).

In contrast, long non-coding RNAs (lncRNAs) regulate LRP1 expression at the chromatin level. In particular, the lncRNA annotated as LRP1-AS, modulates LRP1 transcription through interaction with chromatin-associated proteins such as HMGB2 (Yamanaka et al., 2015). This interaction is proposed to influence local chromatin architecture and transcriptional accessibility at the LRP1 promoter, suggesting a mechanism of transcriptional interference or epigenetic scaffolding.

#### 3.3.2 Indirect ncRNA networks

Other non-coding RNAs implicated in AS may indirectly influence LRP1 expression. ANRIL, MALAT1, miR-103/107, miR-124, and miR-1908 intersect with inflammatory signaling, lipid metabolism, efferocytosis, and smooth muscle cell plasticity. Although direct targeting of LRP1 has not been demonstrated, their regulatory networks overlap with pathways involving NF-κB activation, cholesterol efflux, and metabolic stress responses. Notably, lncRNA-mediated modulation of NF-κB signaling has emerged as an important regulatory mechanism, including in AS, influencing inflammatory gene expression and macrophage activation states (Hussain et al., 2024). The long non-coding RNA LINC00657 has been shown to act as a competing endogenous RNA by sponging miR-590-3p, thereby relieving repression of HIF-1α and promoting angiogenesis in oxLDL-treated endothelial cells (Bao et al., 2018). In parallel, cytokine-driven signaling pathways further interact with ncRNA networks. The TNK1-Tyk2/STAT1 axis has been implicated in inflammatory responses (Bao et al., 2020), supporting the idea of interconnected inflammatory and transcriptional circuits.

Furthermore, while bioinformatic analyses have identified several non-coding RNA candidates potentially targeting LRP1, their regulatory roles remain largely unvalidated, highlighting an important gap in locus-specific functional evidence. Overall, non-coding RNAs constitute the most substantiated epigenetic layer directly or indirectly influencing LRP1 expression. In contrast, chromatin-level regulation of the LRP1 locus by histone modifiers or DNA methylation enzymes remains largely uncharacterized.

These observations further support the hierarchical nature of LRP1 regulation, where non-coding RNA-mediated mechanisms provide stronger direct or functional evidence compared to chromatin-level regulation.

### 3.4 Chromatin architecture and epigenetic regulation: a hierarchical perspective and unresolved gaps

Beyond transcription factor-dependent control, LRP1 expression may be influenced by epigenetic mechanisms that shape chromatin accessibility and transcriptional stability (Figure 1E). Epigenetic regulation, including DNA methylation and histone modifications, has been extensively characterized in AS, where it governs inflammatory activation, lipid handling, and vascular cell plasticity (Khan et al., 2021; Masi et al., 2021; Zhang et al., 2023). However, in contrast to transcription factor-mediated and non-coding RNA-mediated regulation, locus-specific evidence linking these chromatin-level mechanisms to LRP1 expression remains limited.

#### 3.4.1 DNA methylation

DNA methylation represents a central epigenetic mechanism, which is dynamically controlled by DNA methyltransferases. DNMT1 and DNMT3A/B promote hypermethylation of genes involved in oxidative stress and cholesterol efflux, contributing to endothelial dysfunction, macrophage activation, and plaque progression (Gagliardi et al., 2018; Zaina, 2025). Conversely, TET2 maintains anti-inflammatory gene programs and restrains IL-1β-driven inflammation; loss-of-function mutations in TET2 promote clonal hematopoiesis and accelerate AS (Fuster et al., 2017).

Despite the central role of these enzymes in shaping vascular and immune transcriptional programs, no studies have directly assessed DNA methylation patterns at the LRP1 locus, and its contribution to LRP1 transcriptional regulation remains undefined. A systematic overview of DNA methylation enzymes and their roles in AS is provided in Supplementary Tables S3, S4.

#### 3.4.2 Histone modifications

Histone modifications are key regulators of chromatin accessibility and transcriptional activity in vascular and immune cells. Activating marks such as H3K27ac and H3K4me3 are enriched at promoters and enhancers of inflammatory and lipid metabolism genes, whereas repressive marks including H3K27me3 and H3K9me3 contribute to gene silencing during macrophage polarization and VSMC phenotypic transitions (Saeed et al., 2014; Schmidt et al., 2016).

Chromatin-modifying enzymes further shape these processes. Histone acetyltransferases (KAT2A/PCAF, p300/CBP, MYST family members) amplify NF-κB-dependent transcription and contribute to endothelial dysfunction and macrophage activation (Rydberg et al., 2004; Inoue et al., 2007; Fang et al., 2014; Piaszyk-Borychowska et al., 2019). Histone deacetylases, including HDAC1-3, HDAC9, and SIRT family members, exert cell-type-specific effects on inflammatory signaling and plaque stability. Likewise, histone methyltransferases (SUV39H1/2, EZH2, KMT2 family members) and demethylases (KDM families) regulate macrophage polarization, VSMC proliferation, and inflammatory gene expression. Emerging chromatin-associated mechanisms, including metabolite-driven histone modifications such as lactylation, crotonylation, and succinylation, have been proposed to link cellular metabolism with transcriptional regulation in AS (Bao et al., 2024; Zhao et al., 2024). These modifications reflect changes in glycolytic flux, mitochondrial activity, and acyl-CoA availability, and have been associated with inflammatory gene expression and macrophage activation (Saeed et al., 2014; Schmidt et al., 2016; Shu et al., 2025). However, their contribution to LRP1 regulation remains speculative and unsupported by direct experimental evidence.

A comprehensive overview of chromatin-modifying enzymes implicated in atherosclerosis is provided in Supplementary Tables S5–S9, further illustrating the limited availability of LRP1-specific epigenetic data.

Taken together, although these chromatin modifiers regulate pathways that functionally intersect with LRP1 biology, systematic chromatin-level interrogation of LRP1 regulatory regions has not been performed. This highlights a key gap in our understanding of LRP1 transcriptional control and underscores the need for locus-specific epigenomic analyses, particularly using single-cell and integrative multi-omics approaches to resolve cell-type-specific regulatory dynamics.

### 3.5 Genetic variants of LRP1

Genetic variation provides additional evidence supporting enhancer-mediated regulation of LRP1 (Figure 1F). Recent studies have identified both genetic variants and distal regulatory elements that contribute to the control of LRP1 expression in VSMC. The cardiovascular risk variant rs11172113 resides in an intronic enhancer of the LRP1 locus that recruits the transcriptional repressor SNAIL (SNAI1) in an allele-specific manner, reducing LRP1 expression and altering extracellular matrix remodeling programs (Liu et al., 2023; 2024). In addition, MECP2 (Methyl-CpG-binding protein 2) has been implicated in modulating enhancer activity at this locus, supporting a role for chromatin-dependent regulation of LRP1 transcription. Additional genetic variants within the LRP1 locus, including rs1799986 (Grimmer et al., 2014), rs1800137 (Lee et al., 2017), and rs1466535 (Galora et al., 2015), have been associated with altered receptor regulation or disease susceptibility in non-atherosclerotic contexts.

Overall, current evidence suggests that regulatory architecture, rather than coding mutations, may underlie disease-associated modulation of LRP1 expression. However, systematic integration of genetic, epigenomic, and enhancer-mapping data at the LRP1 locus has not yet been achieved, limiting the ability to define its regulatory landscape with precision.

## 4 Final perspectives and conclusion

LRP1 functions as a key integrator of lipid metabolism, receptor trafficking, and inflammatory signaling in vascular and immune cells, with a role that varies across cellular contexts and disease stages. Experimental evidence supports a context-dependent contribution of LRP1 in macrophage activation, foam cell formation, and vascular remodeling (Yancey et al., 2011; Solanelles Curco et al., 2025). Collectively, current evidence supports a hierarchical model of regulation in which transcriptional and post-transcriptional mechanisms, including SREBPs, HIF-1α, and non-coding RNAs, provide the most direct and substantiated control over LRP1 expression. In contrast, although chromatin-associated processes broadly shape inflammatory and metabolic gene programs, their direct, locus-specific role in LRP1 regulation remains largely unresolved.

Within this framework, the apparently divergent roles of LRP1 in atherosclerosis are best explained by differences in cellular context, ligand availability, and post-translational regulation, particularly receptor abundance and proteolytic processing, rather than by intrinsically opposing functions.

Future research should prioritize three areas: (i) locus-specific epigenomic profiling of the LRP1 regulatory landscape; (ii) integration of transcriptional, post-transcriptional, and chromatin-level data through single-cell multi-omics approaches; and (iii) functional validation of regulatory mechanisms across vascular cell types under disease-relevant conditions. Addressing these questions will be essential to define how regulatory states shape LRP1-dependent functions and to evaluate its potential as a biomarker or therapeutic target in atherosclerosis.
