# Supplementary material for: LRP1 in atherosclerosis: a hierarchical view of regulatory mechanisms and epigenetic knowledge gaps
Source: Front Cell Dev Biol. 2026 May 8;14:1829891. doi: 10.3389/fcell.2026.1829891 (PMC13194561; doi:10.3389/fcell.2026.1829891)
Supplement: Supplementary file 1 [file DataSheet1.pdf]

Table S1: Transcriptional regulation of LRP1

Transcription factor	Cell type	Stimulus/Context	Effect on LRP1	Mechanism	Evidence level
SREBP-2	VSMC	Aggregated LDL	↑	Reduced promoter binding repression	Direct
SREBP-1	Macrophages	LPS / inflammation	↓	Increased transcriptional repression	Direct
HIF-1α	VSMC	Hypoxia	↑	Binding to hypoxia response elements	Direct
CREB	Vascular cells	Inflammatory signaling	Potential ↑	Upstream transcriptional regulation	Direct
NF-κB	Macrophages	TLR activation	Potential modulation	Inflammatory transcriptional programs	Indirect (contextual)
PPARs	Macrophages/VSMC	Lipid metabolism	Potential modulation	Metabolic transcriptional control	Indirect (contextual)
STATs	Immune cells	Cytokine signaling	Potential modulation	Cytokine-driven transcription	Indirect (contextual)
KLFs	Vascular cells	Shear stress / inflammation	Potential modulation	Vascular homeostasis regulation	Indirect (contextual)

Table S2. Non-coding RNAs involved in atherosclerosis and their relevance to LRP1					
**ncRNA**	**Type**	**Role in AS (summary)**	**Effect on LRP1**	**Mechanism**	**Evidence level**
miR-205-5p	miRNA	Promotes lipid accumulation, oxidative stress, and plaque instability (Huang et al., 2022).	↓	Direct binding to LRP1 3′UTR, impairing LRP1/ABCA1 axis	Direct
miR-429	miRNA	Promotes endothelial dysfunction and inflammatory signaling (Zhang et al., 2015)	↓	Represses LRP1 expression in endothelial cells	Direct
LRP1-AS	lncRNA	Regulates lipid metabolism and vascular inflammation (Yamanaka et al., 2015)	Modulates	Chromatin-associated regulation via HMGB2	Direct
miR-126-3p	miRNA	Atheroprotective; regulates endothelial activation and angiogenesis (Guo et al., 2025; Woźniak et al., 2025)	Indirect	Targets LRP6 (Wnt pathway overlap)	Indirect
ANRIL	lncRNA	Promotes proliferation and inflammation via NF-κB and TGF-β pathways (Razeghian-Jahromi et al., 2022)	Indirect	Modulates inflammatory signaling networks	Indirect
MALAT1	lncRNA	Enhances endothelial dysfunction and macrophage activation (Huang et al., 2018; Zhu et al., 2019)	Indirect	NF-κB-dependent pathways	Indirect
miR-103	miRNA	Promotes inflammation and VSMC calcification (Jiang et al., 2020; He et al., 2021)	Indirect	PTEN/AKT pathway overlap	Indirect
miR-107	miRNA	Anti-inflammatory and endothelial protective (Gao et al., 2019)	Indirect	HMGB1-related pathways	Indirect
miR-199a-3p/5p	miRNA	Regulates apoptosis, autophagy, and inflammation (González-López et al., 2023)	Indirect	SIRT1/NF-κB modulation	Indirect
miR-1908	miRNA	Regulates lipid homeostasis via LDLR (Beehler et al., 2021; Soubeyrand et al., 2021)	Indirect	Lipid receptor network overlap	Indirect
miR-124	miRNA	Anti-inflammatory; regulates macrophage polarization (Liang et al., 2020)	Indirect	p38/STAT3 pathway	Indirect
APOA1-AS	lncRNA	Impairs HDL biogenesis and cholesterol efflux (Sri et al., 2025)	Indirect	Histone methylation of APOA1	Indirect

Table S3: DNA Methylation Machinery (DNMTs)			
**Enzyme**	**Role in AS (summary)**	**Relevance to LRP1**	**Evidence level**
DNMT1	Promotes endothelial dysfunction, macrophage inflammation, and foam cell formation (Yu et al., 2016; Tang et al., 2019; Zaina, 2025).	Indirect	Indirect
DNMT3A	Regulates macrophage polarization and clonal hematopoiesis-driven inflammation (F Yang et al., 2022b)	Not established	Not investigated
DNMT3B	Promotes inflammatory gene expression under oxidative stress (Gagliardi et al., 2018; Chen et al., 2024b)	Not established	Not investigated
DNMT3L	Cofactor for DNMT3A/B; limited vascular evidence (Yan et al., 2026)	Not established	Not investigated

Table S4: DNA Demethylation Enzymes (TETs)			
**Enzyme**	**Role in AS (summary)**	**Relevance to LRP1**	**Evidence level**
TET1	Regulates endothelial homeostasis and inflammatory gene expresión (Jin et al., 2014; Weber et al., 2016)	Not established	Not investigated
TET2	Suppresses IL-1β/NLRP3 signaling; clonal hematopoiesis accelerates AS (Fuster et al., 2017)	Not established	Not investigated
TET3	Modulates inflammatory and metabolic transcriptional programs (Jin et al., 2014; Jiang et al., 2018)	Not established	Not investigated

Table S5: Histone Acetylation Writers (HATs)			
**Enzyme**	**Role in AS (summary)**	**Relevance to LRP1**	**Evidence level**
KAT2A / PCAF (GNAT)	Promote NF-κB-dependent inflammation and endothelial dysfunction (Rydberg et al., 2004; Yang et al., 2022b).	Not established	Not investigated
MYST family (TIP60, MOF, HBO1, MOZ, MORF)	Regulate chromatin accessibility, oxidative stress, and lipid metabolism (Piaszyk-Borychowska et al., 2019) (Liu et al., 2017; Jiang et al., 2022)..	Not established	Not investigated
p300 / CBP	Amplify inflammatory transcription and interact with metabolic regulators (e.g., SREBPs) (Fang et al., 2014; Zhang et al., 2023a)	Indirect	Indirect
CLOCK / BRCA2	Circadian and genomic stability roles; limited AS-specific epigenetic relevance (Gorodetska et al., 2019)	Not established	Not investigated

Table S6: Histone Acetylation Erasers (HDACs / Sirtuins)			
**Enzyme**	**Role in AS (summary)**	**Relevance to LRP1**	**Evidence level**
Class I HDACs (HDAC1–3, 8)	Regulate endothelial inflammation and macrophage activation (Spin et al., 2012; Hoeksema et al., 2014; Chen et al., 2023).	Indirect	Indirect
Class IIa HDACs (HDAC4–5, 7, 9)	Control VSMC remodeling and inflammatory signaling (HDAC9 strongly pro-atherogenic) (Cao et al., 2014; Chen et al., 2020)	Indirect	Indirect
Sirtuins (SIRT1–7)	Link metabolism, oxidative stress, and inflammation; largely atheroprotective (D'Onofrio et al., 2018; Toulassi et al., 2021).	Indirect	Indirect
HDAC11	Promotes pro-inflammatory macrophage polarization (Luan et al., 2022)	Not established	Not investigated

Table S7: Histone Methylation Writers (KMTs)			
**Enzyme**	**Role in AS (summary)**	**Relevance to LRP1**	**Evidence level**
SUV39H1/2	Repress inflammatory genes; promote plaque stability (Jiang et al., 2018; Weirich et al., 2021).	Not established	Not investigated
G9a / GLP	Promote inflammatory activation and foam cell formation (Jiang et al., 2018; Weirich et al., 2021).	Not established	Not investigated
SETDB1	Limits macrophage activation and vascular inflammation (Hachiya et al., 2016)	Not established	Not investigated
EZH2	Strongly pro-atherogenic; promotes inflammation and VSMC dedifferentiation (Neele et al., 2021).	Not established	Not investigated
DOT1L	Enhances endothelial inflammation and adhesion molecule expression (Willemsen et al., 2022)	Not established	Not investigated
KMT2 family (MLL)	Regulate enhancer activation and inflammatory transcription (Ang et al., 2016)	Not established	Not investigated
SMYD2 / SMYD3	Modulate NF-κB signaling and cell proliferation (Zhou et al., 2023)	Not established	Not investigated

Table S8: Histone Demethylases (KDMs)			
**Enzyme**	**Role in AS (summary)**	**Relevance to LRP1**	**Evidence level**
KDM1A (LSD1)	Promotes inflammatory activation and foam cell formation (Jiang et al., 2018; Manea et al., 2022)	Indirect	Indirect
KDM2A/B	Regulate inflammatory gene repression and endothelial activation (Ma and Zhang, 2024)	Not established	Not investigated
KDM3A/B	Promote endothelial inflammation and macrophage activation (Ma and Zhang, 2024).	Not established	Not investigated
KDM4 family	Enhance inflammatory gene expression and VSMC proliferation (Jiang et al., 2018)	Not established	Not investigated
KDM5 family	Regulate promoter activity and inflammatory gene repression (Zhang et al., 2023b; Chen et al., 2024a).	Not established	Not investigated
KDM6A/B	Strong drivers of inflammatory activation and macrophage polarization (Jiang et al., 2018)	Not established	Not investigated
KDM7A/B	Regulate endothelial inflammatory gene activation (Higashijima et al., 2020; Qu et al., 2023).	Not established	Not investigated

Table S9. Additional chromatin and epigenetic regulators in atherosclerosis			
**Enzyme / Process**	**Role in AS (summary)**	**Relevance to LRP1**	**Evidence level**
DNA repair enzymes (TDG, UNG, SMUG1, AAG)	Maintain genomic integrity under oxidative stress (Mahmoudi et al., 2006; Doseth et al., 2011; Calvo et al., 2013; Onabote et al., 2022)	Not established	Not investigated
ALKBH family	Regulate mitochondrial function and oxidative stress (Jingushi et al., 2021; Luo et al., 2022)	Indirect	Indirect
Aurora B / MSK1/2	Histone phosphorylation; promote proliferation and inflammation (Hirota et al., 2005)	Indirect	Indirect
JAK2 / PIM1	Regulate cytokine signaling and vascular remodeling (Dotan et al., 2022; Xue et al., 2025)	Indirect	Indirect
PP1 / PP2A	Control chromatin dynamics and macrophage polarization (Dotan et al., 2022; Li et al., 2022)	Indirect	Indirect
RNF20/40 / RING1B	Histone ubiquitination and transcriptional regulation (Eskeland et al., 2010; Tarcic et al., 2016)	Not established	Not investigated
Metabolic chromatin marks (lactylation, crotonylation, etc.)	Link metabolism to gene regulation (Franck et al., 2018; Bao et al., 2024; Zhao et al., 2024)	Not established	Not investigated

Bibliography

Ang, S.-Y., Uebersohn, A., Spencer, C. I., Huang, Y., Lee, J.-E., Ge, K., et al. (2016). KMT2D regulates specific programs in heart development via histone H3 lysine 4 di-methylation. *Development* 143, 810–821. doi: 10.1242/dev.132688

Bao, C., Ma, Q., Ying, X., Wang, F., Hou, Y., Wang, D., et al. (2024). Histone lactylation in macrophage biology and disease: from plasticity regulation to therapeutic implications. *eBioMedicine* 111, 105502. doi: 10.1016/j.ebiom.2024.105502

Beehler, K., Nikpay, M., Lau, P., Dang, A.-T., Lagace, T. A., Soubeyrand, S., et al. (2021). A Common Polymorphism in the FADS1 Locus Links miR1908 to Low-Density Lipoprotein Cholesterol Through BMP1. *Arterioscler Thromb Vasc Biol* 41, 2252–2262. doi: 10.1161/ATVBAHA.121.316473

Calvo, J. A., Moroski-Erkul, C. A., Lake, A., Eichinger, L. W., Shah, D., Jhun, I., et al. (2013). Aag DNA Glycosylase Promotes Alkylation-Induced Tissue Damage Mediated by Parp1. *PLoS Genet* 9, e1003413. doi: 10.1371/journal.pgen.1003413

Cao, Q., Rong, S., Repa, J. J., St Clair, R., Parks, J. S., and Mishra, N. (2014). Histone deacetylase 9 represses cholesterol efflux and alternatively activated macrophages in atherosclerosis development. *Arterioscler Thromb Vasc Biol* 34, 1871–1879. doi: 10.1161/ATVBAHA.114.303393

Chen, H., Sarah, L., Pucciarelli, D., Mao, Y., Diolaiti, M. E., Fujimori, D. G., et al. (2024a). Histone demethylase enzymes KDM5A and KDM5B modulate immune response by suppressing transcription of endogenous retroviral elements. *bioRxiv*, 2024.09.23.614494. doi: 10.1101/2024.09.23.614494

Chen, J., Yang, X., Li, Q., Ma, J., Li, H., Wang, L., et al. (2024b). Inhibiting DNA methyltransferase DNMT3B confers protection against ferroptosis in nucleus pulposus and ameliorates intervertebral disc degeneration via upregulating SLC40A1. *Free Radical Biology and Medicine* 220, 139–153. doi: 10.1016/j.freeradbiomed.2024.05.007

Chen, L., He, J., Zhang, Y., Li, Y., Zhang, T., Wang, R., et al. (2023). Regulation of endothelial-to-mesenchymal transition by histone deacetylase 3 posttranslational modifications in neointimal hyperplasia. *Ann Transl Med* 11, 207–207. doi: 10.21037/atm-22-4371

Chen, X., He, Y., Fu, W., Sahebkar, A., Tan, Y., Xu, S., et al. (2020). Histone Deacetylases (HDACs) and Atherosclerosis: A Mechanistic and Pharmacological Review. *Front Cell Dev Biol* 8, 581015. doi: 10.3389/fcell.2020.581015

D'Onofrio, N., Servillo, L., and Balestrieri, M. L. (2018). SIRT1 and SIRT6 Signaling Pathways in Cardiovascular Disease Protection. *Antioxidants & Redox Signaling* 28, 711–732. doi: 10.1089/ars.2017.7178

Doseth, B., Visnes, T., Wallenius, A., Ericsson, I., Sarno, A., Pettersen, H. S., et al. (2011). Uracil-DNA Glycosylase in Base Excision Repair and Adaptive Immunity. *J Biol Chem* 286, 16669–16680. doi: 10.1074/jbc.M111.230052

Dotan, I., Yang, J., Ikeda, J., Roth, Z., Pollock-Tahiri, E., Desai, H., et al. (2022). Macrophage Jak2 deficiency accelerates atherosclerosis through defects in cholesterol efflux. *Commun Biol* 5, 132. doi: 10.1038/s42003-022-03078-5

Eskeland, R., Leeb, M., Grimes, G. R., Kress, C., Boyle, S., Sproul, D., et al. (2010). Ring1B Compacts Chromatin Structure and Represses Gene Expression Independent of Histone Ubiquitination. *Mol Cell* 38, 452–464. doi: 10.1016/j.molcel.2010.02.032

Fang, F., Xu, Y., Chew, K.-K., Chen, X., Ng, H.-H., and Matsudaira, P. (2014). Coactivators p300 and CBP Maintain the Identity of Mouse Embryonic Stem Cells by Mediating Long-Range Chromatin Structure. *Stem Cells* 32, 1805–1816. doi: 10.1002/stem.1705

Franck, G., Mawson, T. L., Folco, E. J., Molinaro, R., Ruvkun, V., Engelbertsen, D., et al. (2018). Roles of PAD4 and NETosis in Experimental Atherosclerosis and Arterial Injury: Implications for Superficial Erosion. *Circ Res* 123, 33–42. doi: 10.1161/CIRCRESAHA.117.312494

Fuster, J. J., MacLauchlan, S., Zuriaga, M. A., Polackal, M. N., Ostriker, A. C., Chakraborty, R., et al. (2017). Clonal hematopoiesis associated with TET2 deficiency accelerates atherosclerosis development in mice. *Science* 355, 842–847. doi: 10.1126/science.aag1381

Gagliardi, M., Strazzullo, M., and Matarazzo, M. R. (2018). DNMT3B Functions: Novel Insights From Human Disease. *Front Cell Dev Biol* 6, 140. doi: 10.3389/fcell.2018.00140

Gao, Z.-F., Ji, X.-L., Gu, J., Wang, X.-Y., Ding, L., and Zhang, H. (2019). microRNA-107 protects against inflammation and endoplasmic reticulum stress of vascular endothelial cells via KRT1-dependent Notch signaling pathway in a mouse model of coronary atherosclerosis. *J Cell Physiol* 234, 12029–12041. doi: 10.1002/jcp.27864

González-López, P., Álvarez-Villarreal, M., Ruiz-Simón, R., López-Pastor, A. R., de Ceniga, M. V., Esparza, L., et al. (2023). Role of miR-15a-5p and miR-199a-3p in the inflammatory pathway regulated by NF-κB in experimental and human atherosclerosis. *Clinical and Translational Medicine* 13, e1363. doi: 10.1002/ctm2.1363

Gorodetska, I., Kozeretska, I., and Dubrovska, A. (2019). BRCA Genes: The Role in Genome Stability, Cancer Stemness and Therapy Resistance. *J Cancer* 10, 2109–2127. doi: 10.7150/jca.30410

Guo, B., Gu, J., Zhuang, T., Zhang, J., Fan, C., Li, Y., et al. (2025). MicroRNA-126: From biology to therapeutics. *Biomedicine & Pharmacotherapy* 185, 117953. doi: 10.1016/j.biopha.2025.117953

Hachiya, R., Shiihashi, T., Shirakawa, I., Iwasaki, Y., Matsumura, Y., Oishi, Y., et al. (2016). The H3K9 methyltransferase Setdb1 regulates TLR4-mediated inflammatory responses in macrophages. *Sci Rep* 6, 28845. doi: 10.1038/srep28845

He, L., Xu, J., Bai, Y., Zhang, H., Zhou, W., Cheng, M., et al. (2021). MicroRNA-103a regulates the calcification of vascular smooth muscle cells by targeting runt-related transcription factor 2 in high phosphorus conditions. *Exp Ther Med* 22, 1036. doi: 10.3892/etm.2021.10468

Higashijima, Y., Matsui, Y., Shimamura, T., Nakaki, R., Nagai, N., Tsutsumi, S., et al. (2020). Coordinated demethylation of H3K9 and H3K27 is required for rapid inflammatory responses of endothelial cells. *The EMBO Journal* 39, e103949. doi: 10.15252/embj.2019103949

Hirota, T., Lipp, J. J., Toh, B.-H., and Peters, J.-M. (2005). Histone H3 serine 10 phosphorylation by Aurora B causes HP1 dissociation from heterochromatin. *Nature* 438, 1176–1180. doi: 10.1038/nature04254

Hoeksema, M. A., Gijbels, M. J., Van Den Bossche, J., Van Der Velden, S., Sijm, A., Neele, A. E., et al. (2014). Targeting macrophage Histone deacetylase 3 stabilizes atherosclerotic lesions. *EMBO Mol Med* 6, 1124–1132. doi: 10.15252/emmm.201404170

Huang, P., Zhang, Y., Wang, F., Qin, M., and Ren, L. (2022). MiRNA-205–5p regulates the ERBB4/AKT signaling pathway to inhibit the proliferation and migration of HAVSMCs induced by ox-LDL. *Pathology - Research and Practice* 233, 153858. doi: 10.1016/j.prp.2022.153858

Jiang, L., Qiao, Y., Wang, Z., Ma, X., Wang, H., and Li, J. (2020). Inhibition of microRNA-103 attenuates inflammation and endoplasmic reticulum stress in atherosclerosis through disrupting the PTEN-mediated MAPK signaling. *J Cell Physiol* 235, 380–393. doi: 10.1002/jcp.28979

Jiang, L.-P., Yu, X.-H., Chen, J.-Z., Hu, M., Zhang, Y.-K., Lin, H.-L., et al. (2022). Histone Deacetylase 3: A Potential Therapeutic Target for Atherosclerosis. *Aging and disease* 13, 773. doi: 10.14336/AD.2021.1116

Jiang, W., Agrawal, D. K., and Boosani, C. S. (2018). Cell-specific histone modifications in atherosclerosis (Review). *Mol Med Rep* 18, 1215–1224. doi: 10.3892/mmr.2018.9142

Jin, C., Lu, Y., Jelinek, J., Liang, S., Estecio, M. R. H., Barton, M. C., et al. (2014). TET1 is a maintenance DNA demethylase that prevents methylation spreading in differentiated cells. *Nucleic Acids Res* 42, 6956–6971. doi: 10.1093/nar/gku372

Jin, F., Li, J., Guo, J., Doeppner, T. R., Hermann, D. M., Yao, G., et al. (2021). Targeting epigenetic modifiers to reprogramme macrophages in non-resolving inflammation-driven atherosclerosis. *Eur Heart J Open* 1, oeab022. doi: 10.1093/ehjopen/oeab022

Jingushi, K., Aoki, M., Ueda, K., Kogaki, T., Tanimoto, M., Monoe, Y., et al. (2021). ALKBH4 promotes tumourigenesis with a poor prognosis in non-small-cell lung cancer. *Sci Rep* 11, 8677. doi: 10.1038/s41598-021-87763-1

Li, R., Zhang, C., Xie, F., Zhou, X., Hu, X., Shi, J., et al. (2022). Protein Phosphatase 2A Deficiency in Macrophages Increases Foam Cell Formation and Accelerates Atherosclerotic Lesion Development. *Front Cardiovasc Med* 8, 745009. doi: 10.3389/fcvm.2021.745009

Liang, X., Wang, L., Wang, M., Liu, Z., Liu, X., Zhang, B., et al. (2020). MicroRNA-124 inhibits macrophage cell apoptosis via targeting p38/MAPK signaling pathway in atherosclerosis development. *Aging (Albany NY)* 12, 13005–13022. doi: 10.18632/aging.103387

Liu, Y., Reynolds, L. M., Ding, J., Hou, L., Lohman, K., Young, T., et al. (2017). Blood monocyte transcriptome and epigenome analyses reveal loci associated with human atherosclerosis. *Nat Commun* 8, 393. doi: 10.1038/s41467-017-00517-4

Luan, Y., Liu, H., Luan, Y., Yang, Y., Yang, J., and Ren, K.-D. (2022). New Insight in HDACs: Potential Therapeutic Targets for the Treatment of Atherosclerosis. *Front Pharmacol* 13, 863677. doi: 10.3389/fphar.2022.863677

Luo, L., Liu, Y., Nizigiyimana, P., Ye, M., Xiao, Y., Guo, Q., et al. (2022). DNA 6mA Demethylase ALKBH1 Orchestrates Fatty Acid Metabolism and Suppresses Diet-Induced Hepatic Steatosis. *Cell Mol Gastroenterol Hepatol* 14, 1213–1233. doi: 10.1016/j.jcmgh.2022.08.011

Ma, H., and Zhang, T. (2024). Histone demethylase KDM3B mediates matrix stiffness-induced osteogenic differentiation of adipose-derived stem cells. *Archives of Biochemistry and Biophysics* 757, 110028. doi: 10.1016/j.abb.2024.110028

Mahmoudi, M., Mercer, J., and Bennett, M. (2006). DNA damage and repair in atherosclerosis. *Cardiovasc Res* 71, 259–268. doi: 10.1016/j.cardiores.2006.03.002

Manea, S.-A., Vlad, M.-L., Lazar, A.-G., Muresian, H., Simionescu, M., and Manea, A. (2022). Pharmacological Inhibition of Lysine-Specific Demethylase 1A Reduces Atherosclerotic Lesion Formation in Apolipoprotein E-Deficient Mice by a Mechanism Involving Decreased Oxidative Stress and Inflammation; Potential Implications in Human Atherosclerosis. *Antioxidants* 11, 2382. doi: 10.3390/antiox11122382

Neele, A. E., Chen, H.-J., Gijbels, M. J. J., Van Der Velden, S., Hoeksema, M. A., Boshuizen, M. C. S., et al. (2021). Myeloid Ezh2 Deficiency Limits Atherosclerosis Development. *Front. Immunol.* 11, 594603. doi: 10.3389/fimmu.2020.594603

Onabote, O., Hassan, H. M., Isovic, M., and Torchia, J. (2022). The Role of Thymine DNA Glycosylase in Transcription, Active DNA Demethylation, and Cancer. *Cancers (Basel)* 14, 765. doi: 10.3390/cancers14030765

Piaszyk-Borychowska, A., Széles, L., Csermely, A., Chiang, H.-C., Wesoły, J., Lee, C.-K., et al. (2019). Signal Integration of IFN-I and IFN-II With TLR4 Involves Sequential Recruitment of STAT1-Complexes and NFκB to Enhance Pro-inflammatory Transcription. *Front. Immunol.* 10, 1253. doi: 10.3389/fimmu.2019.01253

Qu, L., Yin, T., Zhao, Y., Lv, W., Liu, Z., Chen, C., et al. (2023). Histone demethylases in the regulation of immunity and inflammation. *Cell Death Discov* 9, 188. doi: 10.1038/s41420-023-01489-9

Razeghian-Jahromi, I., Karimi Akhormeh, A., and Zibaeenezhad, M. J. (2022). The Role of ANRIL in Atherosclerosis. *Dis Markers* 2022, 8859677. doi: 10.1155/2022/8859677

Rydberg, E. K., Krettek, A., Ullström, C., Ekström, K., Svensson, P.-A., Carlsson, L. M. S., et al. (2004). Hypoxia Increases LDL Oxidation and Expression of 15-Lipoxygenase-2 in Human Macrophages. *ATVB* 24, 2040–2045. doi: 10.1161/01.ATV.0000144951.08072.0b

Soubeyrand, S., Lau, P., Beehler, K., McShane, K., and McPherson, R. (2021). miR1908-5p regulates energy homeostasis in hepatocyte models. *Sci Rep* 11, 23748. doi: 10.1038/s41598-021-03156-4

Spin, J. M., Maegdefessel, L., and Tsao, P. S. (2012). Vascular smooth muscle cell phenotypic plasticity: focus on chromatin remodelling. *Cardiovasc Res* 95, 147–155. doi: 10.1093/cvr/cvs098

Sri, A. A., Veeraraghavan, V. P., Patil, S., and Raj, A. T. (2025). Long Non-Coding RNA as a Potential Diagnostic Tool in Coronary Artery Diseases - A Systematic Review. *Niger J Clin Pract* 28, 1–7. doi: 10.4103/njcp.njcp_256_24

Tang, R.-Z., Zhu, J.-J., Yang, F.-F., Zhang, Y.-P., Xie, S.-A., Liu, Y.-F., et al. (2019). DNA methyltransferase 1 and Krüppel-like factor 4 axis regulates macrophage inflammation and atherosclerosis. *Journal of Molecular and Cellular Cardiology* 128, 11–24. doi: 10.1016/j.yjmcc.2019.01.009

Tarcic, O., Pateras, I. S., Cooks, T., Shema, E., Kanterman, J., Ashkenazi, H., et al. (2016). RNF20 Links Histone H2B Ubiquitylation with Inflammation and Inflammation-Associated Cancer. *Cell Rep* 14, 1462–1476. doi: 10.1016/j.celrep.2016.01.020

Toulassi, I. A., Al Saedi, U. A., Gutlapalli, S. D., Poudel, S., Kondapaneni, V., Zeb, M., et al. (2021). A Paradigm Shift in the Management of Atherosclerosis: Protective Role of Sirtuins in Atherosclerosis. *Cureus* 13, e12735. doi: 10.7759/cureus.12735

Weber, A. R., Krawczyk, C., Robertson, A. B., Kuśnierczyk, A., Vågbø, C. B., Schuermann, D., et al. (2016). Biochemical reconstitution of TET1–TDG–BER-dependent active DNA demethylation reveals a highly coordinated mechanism. *Nat Commun* 7, 10806. doi: 10.1038/ncomms10806

Weirich, S., Khella, M. S., and Jeltsch, A. (2021). Structure, Activity and Function of the Suv39h1 and Suv39h2 Protein Lysine Methyltransferases. *Life (Basel)* 11, 703. doi: 10.3390/life11070703

Willemsen, L., Prange, K. H. M., Neele, A. E., Van Roomen, C. P. A. A., Gijbels, M., Griffith, G. R., et al. (2022). DOT1L regulates lipid biosynthesis and inflammatory responses in macrophages and promotes atherosclerotic plaque stability. *Cell Reports* 41, 111703. doi: 10.1016/j.celrep.2022.111703

Woźniak, O., Mierzejewski, B., and Brzoska, E. (2025). MicroRNA-126: A key regulator of angiogenesis, inflammation, and tumorigenesis — Exploring its multifaceted functions in vascular health and cancer. *Biochimica et Biophysica Acta (BBA) - Molecular Basis of Disease* 1871, 167984. doi: 10.1016/j.bbadis.2025.167984

Xue, Z., Han, M., Sun, T., Wu, Y., Xing, W., Mu, F., et al. (2025). PIM1 instigates endothelial-to-mesenchymal transition to aggravate atherosclerosis. *Theranostics* 15, 745–765. doi: 10.7150/thno.102597

Yamanaka, Y., Faghihi, M. A., Magistri, M., Alvarez-Garcia, O., Lotz, M., and Wahlestedt, C. (2015). Antisense RNA Controls LRP1 Sense Transcript Expression through Interaction with a Chromatin-Associated Protein, HMGB2. *Cell Reports* 11, 967–976. doi: 10.1016/j.celrep.2015.04.011

Yan, Y., Zhou, X. E., Thomas, S. L., Liu, M., Lai, G.-Q., Worden, E. J., et al. (2026). Mechanisms of DNMT3A–3L-mediated de novo DNA methylation on chromatin. *Nat Struct Mol Biol* 33, 171–183. doi: 10.1038/s41594-025-01704-4

Yang, H., Sun, Y., Li, Q., Jin, F., and Dai, Y. (2022a). Diverse Epigenetic Regulations of Macrophages in Atherosclerosis. *Front Cardiovasc Med* 9, 868788. doi: 10.3389/fcvm.2022.868788

Yang, H., Sun, Y., Li, Q., Jin, F., and Dai, Y. (2022b). Diverse Epigenetic Regulations of Macrophages in Atherosclerosis. *Frontiers in Cardiovascular Medicine* 9. doi: 10.3389/fcvm.2022.868788

Yu, J., Qiu, Y., Yang, J., Bian, S., Chen, G., Deng, M., et al. (2016). DNMT1-PPARγ pathway in macrophages regulates chronic inflammation and atherosclerosis development in mice. *Sci Rep* 6, 30053. doi: 10.1038/srep30053

Zaina, S. (2025). The functional significance of vascular DNA hypermethylation in atherosclerosis: a historical perspective. *Front. Pharmacol.* 16, 1562674. doi: 10.3389/fphar.2025.1562674

Zhang, L., Zhu, K., Xu, J., Chen, X., Sheng, C., Zhang, D., et al. (2023a). Acetyltransferases CBP/p300 Control Transcriptional Switch of β-Catenin and Stat1 Promoting Osteoblast Differentiation. *Journal of Bone and Mineral Research* 38, 1885–1899. doi: 10.1002/jbmr.4925

Zhang, T., Tian, F., Wang, J., Jing, J., Zhou, S.-S., and Chen, Y.-D. (2015). Atherosclerosis-Associated Endothelial Cell Apoptosis by MiR-429-Mediated Down Regulation of Bcl-2. *Cell Physiol Biochem* 37, 1421–1430. doi: 10.1159/000438511

Zhang, Y., Gao, Y., Jiang, Y., Ding, Y., Chen, H., Xiang, Y., et al. (2023b). Histone demethylase KDM5B licenses macrophage-mediated inflammatory responses by repressing Nfkbia transcription. *Cell Death Differ* 30, 1279–1292. doi: 10.1038/s41418-023-01136-x

Zhao, H., Han, Y., Zhou, P., Guan, H., and Gao, S. (2024). Protein lysine crotonylation in cellular processions and disease associations. *Genes & Diseases* 11, 101060. doi: 10.1016/j.gendis.2023.06.029

Zhou, Y., Sharma, S., Sun, X., Guan, X., Hou, Y., Yang, Z., et al. (2023). SMYD2 Regulates Vascular Smooth Muscle Cell Phenotypic Switching and Intimal Hyperplasia via Interaction with Myocardin. *Res Sq*, rs.3.rs-2721176. doi: 10.21203/rs.3.rs-2721176/v1
